# Digital reminiscence therapy in dementia care: a systematic review and meta-analysis

**DOI:** 10.1186/s12883-026-04759-y

**Published:** 2026-03-25

**Authors:** Doan-Khanh Tran, Chaur-Jong Hu, Yi-Chun Kuan

**Affiliations:** 1 International Master Program in Medicine, Taipei Medical University, Taipei, Taiwan; 2 Tran Diep Khanh Medical Polyclinic, Ho Chi Minh City, Vietnam; 3 Department of Neurology, Shuang-Ho Hospital, Taipei Medical University, New Taipei City, Taiwan; 4 Department of Neurology, School of Medicine, College of Medicine, Taipei Medical University, Taipei, Taiwan; 5 Taipei Neuroscience Institute, Taipei Medical University, Taipei, Taiwan; 6 Dementia Center, Shuang-Ho Hospital, Taipei Medical University, New Taipei City, Taiwan; 7 Department of Biomedical Engineering, National Taiwan University, Taipei, Taiwan

**Keywords:** Dementia, Mild cognitive impairment, Reminiscence therapy, Digital reminiscence therapy

## Abstract

**Background:**

Dementia is a major neurodegenerative condition with limited treatment options, making non-pharmacological interventions increasingly important. Reminiscence therapy (RT) has shown benefits in dementia care, and its digital formats - delivered via virtual reality, mobile applications, or multimedia - may offer greater personalization, accessibility, and flexibility in clinical practice. However, evidence of their effectiveness remains fragmented and requires systematic synthesis.

**Methods:**

We conducted a systematic review and meta-analysis to evaluate the efficacy of digital RT in dementia and mild cognitive impairment (MCI), with literature searches conducted up to 1 May 2025. Eligible studies included randomized controlled trials (RCTs) and one-arm pre-post studies. Outcomes assessed were cognition, mood, behavioral and psychological symptoms of dementia (BPSD), quality of life (QOL), communication and engagement. RCTs were pooled in meta-analyses, while pre-post studies were narratively synthesized.

**Results:**

Meta-analysis of RCTs suggested a possible improvement in BPSD, although the evidence was based on few studies of limited size and mixed comparators. Personalized and socially engaging formats appeared most associated with favorable outcomes. No significant effects were found for cognition, mood, QOL or communication and engagement, and overall results remained inconclusive due to variability in intervention design, delivery methods, and outcome measures. Narrative synthesis of pre-post studies revealed mixed results across domains, reflecting small samples, diverse digital formats and lack of control groups.

**Conclusions:**

Digital RT may provide limited benefits for managing BPSD, particularly when incorporating personalized and socially engaging elements. However, the overall evidence remains inconclusive and inconsistent across other outcome domains. Larger, multicenter randomized controlled trials using standardized protocols, validated outcome measures, and direct comparisons with both traditional RT and usual care are needed to clarify its therapeutic value and identify the most effective digital formats and target populations.

**Trial registration:**

The review protocol number was registered on PROSPERO (CRD420251039295).

**Supplementary Information:**

The online version contains supplementary material available at 10.1186/s12883-026-04759-y.

## Introduction

Dementia is a clinical syndrome characterized by the progressive decline of memory, cognition, behavior, and the ability to perform daily activities. It is a leading cause of disability and dependence among older adults globally, with substantial physical, psychological, social, and economic consequences for patients, families, caregivers, and society at large [1]. In addition to cognitive impairment, people living with dementia frequently experience behavioral and psychological symptoms (BPSD) and increasing functional dependence. These impairments also render individuals with dementia particularly vulnerable to abuse, neglect, and exploitation, underscoring the importance of supportive, person-centered, and ethically sensitive care approaches [2]. The global number of people living with dementia is projected to reach 75.6 million by 2030 and 135.3 million by 2050 [3]. Currently, there is no cure for dementia, and available treatments primarily focus on managing symptoms [4]. Mild cognitive impairment (MCI) is a transitional stage between normal cognitive aging and dementia, particularly Alzheimer’s disease (AD). Individuals with MCI are at increased risk of progressing to dementia, with an annual conversion rate of approximately 10 to 20% [5].

Given the limited effectiveness of pharmacological treatments for both dementia and MCI, non-pharmacological interventions play a central role in clinical management. A wide range of such interventions has been explored focusing on improving quality of life, managing symptoms and maintaining function through activities and environmental changes. These therapies, including cognitive stimulation therapy, reminiscence therapy (RT), exercise, music and art therapy, occupational therapy, sensory stimulation; create supportive, structured environments to boost well-being and independence without drugs [6].

RT is a psychosocial intervention that encourages individuals to recall and share past experiences, often using sensory prompts such as photographs, music, or familiar objects. RT has been widely used in dementia care and has shown benefits in cognitive function, mood, quality of life (QOL) [7], and communication [8]. RT is believed to work by stimulating preserved long-term (autobiographical) memory with prompts like photos, music, and objects, bypassing damaged short-term memory to facilitate meaningful conversations and emotional connection. This boosts self-esteem, reduces depression, reinforces identity, and improves quality of life by focusing on past strengths rather than current impairments, fostering positive emotions and social engagement [9].

Traditionally, RT has relied on physical materials and facilitator-led discussion, which may be resource-intensive and variable in structure and delivery. In recent years, digital RT has emerged as an extension of traditional RT that uses digital technologies - such as virtual reality (VR), mobile or web applications, and multimedia integration - either as a complement to or replacement for traditional RT. Similar to traditional RT, studies have suggested that digital RT may improve cognitive function, depressive symptoms [10], and QOL [11]. However, the evidence remains fragmented, and a comprehensive synthesis is lacking. Existing reviews have either focused narrowly on traditional RT or virtual reality (VR) interventions, or, when considering digital RT more broadly, have failed to examine all outcome domains - such as BPSD, communication and engagement - or to identify key features like personalization and social interaction that may influence efficacy [8, 12].

Therefore, this study aimed to conduct a systematic review and meta-analysis focusing specifically on digital RT for individuals with dementia or MCI. It evaluated efficacy across multiple outcome domains, including cognition, mood, BPSD, QOL, communication and engagement. Randomized controlled trials (RCTs) were prioritized for quantitative synthesis because they provided stronger causal inference regarding intervention efficacy, whereas one-arm pre-post studies were narratively synthesized to capture emerging evidence and feasibility. In addition, this review compared digital RT with traditional RT and examined which delivery formats or intervention characteristics appeared most promising.

## Materials and methods

This systematic review and meta-analysis was conducted in accordance with Preferred Reporting Items for Systematic Reviews and Meta-Analyses (PRISMA) guidelines [13]. The review protocol number was registered on PROSPERO (CRD420251039295).

### Inclusion criteria

Studies with either a control group (e.g., RCTs, two-arm trials) or pre-post intervention data (one-arm studies) were included. Inclusion criteria were: (1) participants with dementia (AD, vascular dementia, frontotemporal dementia, Lewy body dementia) or MCI; (2) digital RT, defined as a structured reminiscence-based intervention delivered fully or partially through digital means, including virtual reality, interactive 2D platforms, or digital multimedia (e.g., photographs, audio, or video), with reminiscence as a primary therapeutic component; (3) reporting outcomes from controlled comparisons (traditional RT or usual care) or from pre-post intervention data (one-arm studies); (4) reporting at least one of the following outcomes: cognition, mood, BPSD, QOL, communication and engagement.

Exclusion criteria were: (1) absence of control groups or pre-post data; (2) digital RT combined with other interventions without separable results and (3) qualitative outcomes only.

### Search strategy and study selection

A comprehensive search was conducted in PubMed, Cochrane Library, and Embase. For PubMed, both MeSH terms and free-text keywords were used (e.g., “Dementia”[MeSH], “Alzheimer Disease”[MeSH], “Mild Cognitive Impairment”[MeSH], combined with dementia, Alzheimer disease, cognitive decline, reminiscence, life review therapy, narrative therapy, nostalgia, memory training, digital reminiscence). Equivalent controlled vocabulary (Emtree terms) and free-text keywords were applied in Embase, and the Cochrane Library was searched using MeSH and keywords where applicable. In addition, ClinicalTrials.gov was screened for unpublished trials, and the reference lists of related systematic reviews and meta-analyses were manually checked. The last search was performed on May 1st, 2025.

### Data extraction and intervention classification

Extracted data included the author, year, country, participant characteristics (sample size, mean age, gender, dementia type and stage), type, frequency and duration of digital intervention, follow-up length, outcome domains measured. For outcomes, mean difference (MD), standard deviation (SD), standard error (SE), and confidence interval (CI) were collected. When data were missing, study authors were contacted.

In addition to standard study and outcome data, interventions were classified using an a priori taxonomy based on core therapeutic features and delivery format.

First, personalization level was categorized as high or low. Interventions were considered highly personalized if they used individual life materials, such as personal photographs, music, or videos. Interventions were classified as low personalization if they relied primarily on generic, non-individualized content, such as cultural or historical themes.

Second, social engagement level was classified into three categories. Interventions were rated as high social engagement when delivered in group settings or when involving multiple interaction partners, such as both caregivers and facilitators. Medium social engagement referred to facilitated one-to-one delivery, while low social engagement described self-directed interventions without real-time involvement of other people.

Finally, delivery format was categorized according to the primary technology used. Virtual reality (VR) interventions employed immersive virtual environments, typically delivered via head-mounted displays. Interactive 2D platforms included tablet- or computer-based applications that allowed active user interaction. Digital multimedia interventions used non-immersive digital materials, such as photographs, videos, or audio recordings.

### Methodological quality appraisal

For RCTs, the risk of bias was assessed using the Revised Cochrane Risk of Bias Tool (RoB 2) [14], covering five domains including: (1) randomization, (2) deviations from intended intervention, (3) missing outcome data, (4) outcome measurement, and (5) selective reporting. Studies were rated “low risk” if all domains were low; “some concerns” if at least one domain raised concerns but none were high; and “high risk” if any domain was high or multiple concerns lowered confidence. For non-randomized studies, the Methodological Index for Non-Randomized Studies (MINORS) [15] was applied, evaluating eight items: study aim, consecutive inclusion, prospective data collection, appropriate endpoint, unbiased assessment, adequate follow-up, < 5% loss to follow-up, and sample size calculation. Total scores were interpreted as follows: >12, low risk of bias; 9–12, moderate risk; <9, high risk. Disagreements were resolved by discussion and, if necessary, a third reviewer.

Assessment of publication bias using funnel plots or trim-and-fill methods was not undertaken for outcome domains with fewer than 10 studies, as these approaches are not considered reliable with small numbers of trials.

### Outcomes

Cognition and mood were defined as the primary outcomes. Cognition covered both overall cognitive performance (e.g., Mini-mental State Examination (MMSE), St. Louis University Mental Status (SLUMS)) and memory-specific measures (e.g., Memory Alteration Test (MAT), WHO-UCLA Auditory Verbal Learning Test (AVLT)). These outcomes reflected the central role of memory and global function in dementia. Mood referred to emotional state, including both negative aspects such as anxiety or sadness and positive aspects such as enjoyment or relaxation. Tools such as the Geriatric Depression Scale (GDS) and other affect scales were used to measure these outcomes.

Secondary outcomes included BPSD, which assessed behavioral disturbances such as agitation, irritability, or delusions and was typically measured with the Neuropsychiatric Inventory (NPI). QOL captured patients’ general well-being and functional status. Communication and engagement outcomes encompassed social interaction, participation or engagement in activities, and apathy-related behaviors.

### Statistical analysis

Analyses were conducted in R (version 4.5.1). MD was calculated when outcomes used the same scale; standardized mean difference (SMD) was used when different validated instruments assessed the same outcome. Only RCTs were pooled to maximize causal inference. One-arm pre-post studies were narratively synthesized due to lack of comparators and higher risk of bias. Random-effects models were applied to account for between-study heterogeneity. Heterogeneity was assessed with Cochran’s Q and I² statistic, with thresholds of 25%, 50%, and 75% representing low, moderate, and high heterogeneity, respectively.

When multiple publications arose from the same randomized controlled trial, these reports were treated as a single study. Data from the primary trial report were used for quantitative synthesis, while secondary publications reporting additional intervention-group outcomes were summarized narratively only to avoid double counting of participants.

Pre-specified subgroup analyses explored heterogeneity by intervention type (VR, 2D interactive platform and digital multimedia), personalization level (high and low) and social engagement level (high, medium and low). Comparator type was explicitly examined to distinguish comparisons against an active intervention (traditional RT) from those against usual care, recognizing that active comparators may attenuate observed effect sizes. Intervention intensity was predefined as number of sessions, session duration, and total planned exposure (sessions × minutes); exploratory dose-effect analyses were planned where data permitted. Subgroups with fewer than two studies were not analyzed. Sensitivity analyses were performed by excluding studies at high risk of bias.

For continuous outcomes, change-from-baseline values were prioritized where available. When change-from-baseline data were not reported, post-intervention values were used provided that baseline characteristics between intervention and control groups were comparable.

Influence diagnostics were assessed using leave-one-out analyses, sequentially omitting each study to evaluate the robustness of pooled effect estimates, and were performed only for meta-analyses including more than two studies, as leave-one-out procedures are not informative when only two studies are available.

### Certainty of evidence assessment

The certainty of evidence for each outcome domain was assessed using the GRADE (Grading of Recommendations Assessment, Development and Evaluation) framework. This method considered five domains: risk of bias, inconsistency, indirectness, imprecision, and publication bias. Only data from RCTs were assessed. Evidence started at high certainty and was downgraded as appropriate to moderate, low, or very low.

## Results

### Study selection

**Fig. 1:**
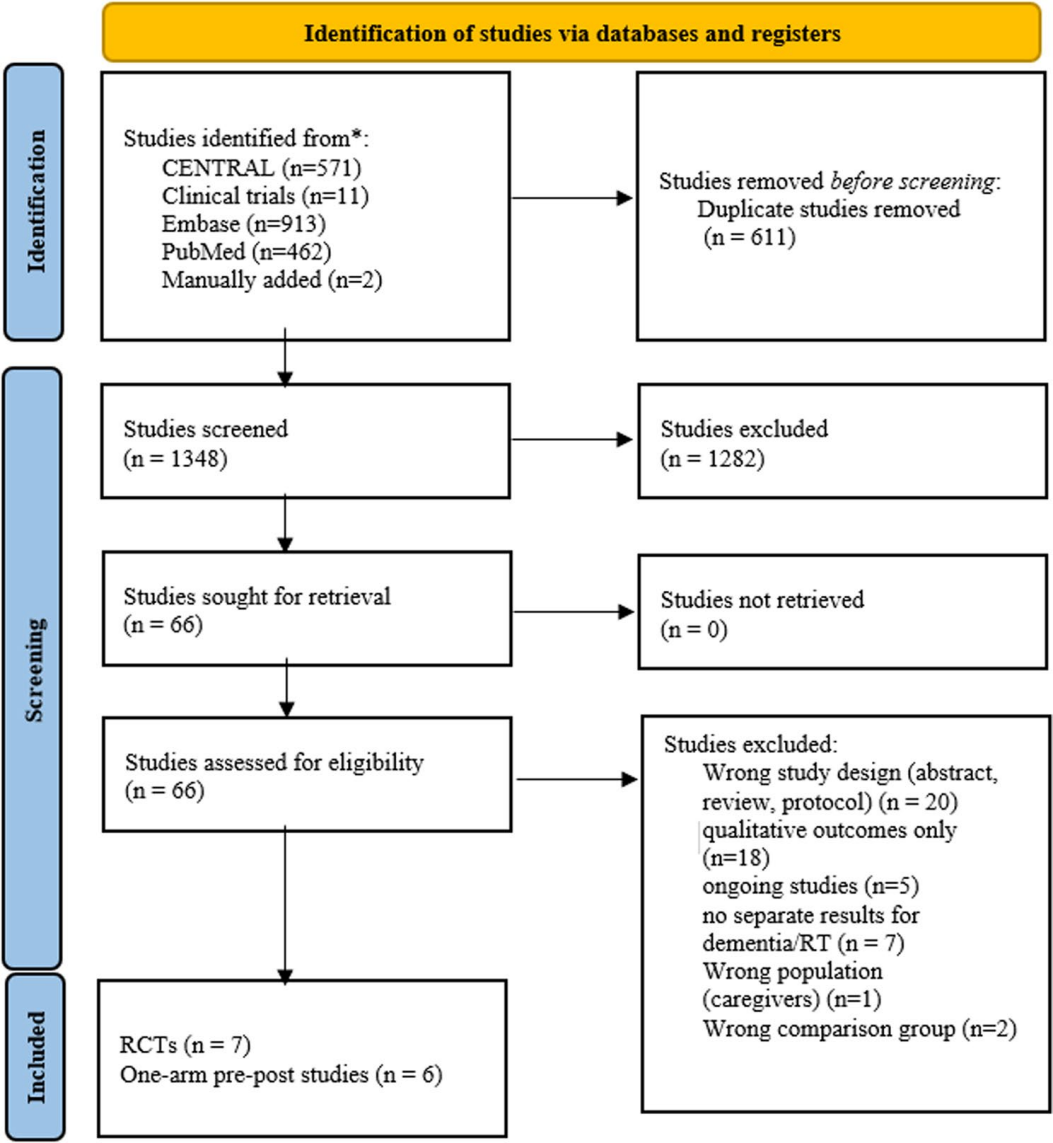
Study search and selection flowchart

Of 1,959 studies identified from Cochrane Library (CENTRAL), Embase, PubMed, ClinicalTrials.gov, and related reviews, 611 duplicates were removed, leaving 1,348 for screening. 66 reports underwent full-text review, and 13 studies [11, 16–28] met the selection criteria: 7 RCTs [11, 16, 20, 21, 24, 25, 28] and 6 one-arm pre-post [18, 19, 22, 23, 26, 27] (Fig. 1). 6 RCTs were included in the meta-analysis; 1 RCT and all pre-post studies contributed to narrative synthesis due to insufficient or incompatible data. One of the included RCTs was published in two reports: the primary publication [21] and a secondary report analyzing only the intervention group [17]. For this review, data from the primary report were included in the RCT meta-analyses, while the secondary report was summarized narratively only, ensuring that participants were not double-counted.

### Study characteristics

Table 1 summarizes the included studies, involving 718 participants: 469 participants from 7 RCTs and 249 participants from 6 one-arm pre-post studies. Participants presented with a range of dementia severity. Most trials recruited individuals with mild to moderate dementia, although some included those with very mild or moderate to severe stages. Where reported, baseline MMSE scores ranged from about 15 to 26, indicating substantial variability in cognitive status across studies.

**Table 1:** Summary of meta-analysis results and GRADE assessment

Author/ Year/ Country	Participants				Intervention		Frequency/ duration of intervention	Follow-up length	Outcome domains assessed
	Sample size	Mean age (SD)	% F	Status	Experiment	Control			
RCTs									
Moon and Park 2020, Korea [21]	41	83.46 (6.06)	100%	Moderate dementia	Interactive 2D platforms: One-on-one reminiscence via Android tablet app with personalized media (photos, music, video) based on family input	One-on-one traditional RT (storytelling on same themes, no digital material)	2 sessions/week for 4 weeks; 30 min/session	Baseline, 2 weeks, 4 weeks (post), and 8 weeks (4-week follow-up)	Cognition, mood, BPSD, communication and engagement
Tominari 2021, Japan [11]	52	Intervention: 85.10, Control: 87.00	Intervention: 19%, Control: 21%	clinically diagnosed with dementia or the MMSE scores ranging 22–26	Virtual reality: Panoramic VR photos (e.g., hometowns, schools) via HMD; group discussion with trained staff	Traditional RT with printed panoramic photos of familiar places	1 session/week × 8 weeks; 30–45 min/session	Baseline and post-intervention	Cognition, mood, BPSD, communication and engagement
Elfrink 2021, Netherlands [20]	42	80.00 (9.40)	55.8%	very mild dementia	Interactive 2D platforms: Online Life Story Book with digital timeline apps (Hellomydear, Albelli), guided by trained volunteers	Care as usual	5 sessions over 8–10 weeks; flexible duration (~ 1–2 h total setup)	Baseline, 3 months, and 6 months	BPSD, communication and engagement
Yu 2019, USA [24]	80 (32 individual, 32 group, 16 control)	82.10 (7.80)	58%	dementia with average baseline MMSE 17.5 ± 6.7	Interactive 2D platforms: Memory Matters reminiscence app	Care as usual	2 sessions/week × 6 weeks (guided), followed by 6 weeks of encouraged self-play	Baseline, 6 weeks (post-intervention), and 12 weeks (after self-play)	BPSD, QOL, communication and engagement
Manav 2019, Turkey [25]	32	74.44 (4.48)	43.8%	mild dementia	Digital multimedia: Group reminiscence with YouTube life-phase videos and traditional memory cues (photos, objects)	Casual conversation, 25–30 min/session, 1×/week × 12 weeks	60 min/session, 1×/week × 12 weeks	Baseline and post-intervention	Cognition, communication and engagement
E. Perez-Saez 2021, Portugal [16]	148	82.53 (7.43)	70.3%	AD or vascular dementia	Digital multimedia: Individual RT program using image cards, stories from Book of the Past and Present, and digital materials	Care as usual	2 sessions/week × 13 weeks; 50 min/session	Baseline and post-intervention	Cognition, mood, QOL
Zhao and Zhang, 2018, China [28]	74	Intervention 76.53 (2.97), Control 77.53 (2.84)	45.9%	Mild to severe dementia	Digital multimedia: Personalized multimedia (photos, videos, music) based on nostalgic themes (childhood, school days, festivals, family life), developed with family/caregiver input	Care as usual	2 sessions/week × 4 weeks; 40–60 min/session	Baseline and post-intervention	Cognition
One-arm Pre-post studies									
Abdalrahim. et al., 2022, UK [19]	60	66.90 (7.40)	51.7%	Mild to severe dementia	Interactive 2D platforms: 10 digital RT sessions (photos, videos, audio) on touch-screen app	N/A	2 sessions/week × 5 weeks; up to 60 min/session	Baseline, post-intervention	Cognition, mood, QOL, communication and engagement
Francis et al., 2020, UK [22]	11	89.30 (range 72–79)	90.9%	AD, vascular dementia, mixed AD and vascular dementia, Parkinson’s type dementia, unspecified dementia	Digital multimedia: 30-min personalized biographical film, developed through 3–4 planning sessions with family and care staff	N/A	≥ 2 sessions/week from Week 8 to Week 32 (~ 24 weeks total)	Baseline, mid-intervention (Week 20), post-intervention (Week 32)	BPSD, QOL
Coelho 2020, Portugal [23]	9	85.60 (7.40)	66.7%	Moderate to severe dementia	Virtual reality: Personalized 360° videos of meaningful locations, viewed via VR headsets	N/A	4 sessions over 2 weeks; 30–60 min/session	Baseline, post-intervention	BPSD, QOL
Astell 2018, UK [26]	143	84.43	82.5%	Dementia	Interactive 2D platforms: CIRCA (Computer Interactive Reminiscence and Conversation Aid) via stand-alone and web-based versions	N/A	2 sessions/week × 4 weeks; 60 min/session	Baseline, post-intervention, and 3 months	
Subramaniam 2016, UK [27]	6	82.20 (range 73–89)	66.7	Mild to moderate dementia	Digital multimedia: Personalized digital life storybook (movie format), co-designed with participants using music, narration, and video	N/A	Participant access for 4 weeks	Baseline, after conventional life storybook, and 4 weeks after digital life storybook introduction	Cognition, mood, QOL
Huang and Yang 2022, Taiwan [18]	20	79.00 (7.80)	55%	mild-moderate dementia (MMSE mean = 15.4)	Virtual reality: Immersive VR reminiscence via HMD, featuring interactive historic scenes with personalized content	N/A	2 sessions/week × 3 months; 10–12 min/session	Baseline, immediately post-intervention, and 3–6 months	Cognition, mood

As shown in Table 1, three main types of digital reminiscence therapy were evaluated. Digital multimedia interventions (5 studies) [16, 22, 25, 27, 28] used photos, videos, music or biographical films, often developed with input from family members or caregivers. Interactive 2D platforms (5 studies) [19–21, 24, 26] involved custom applications on tablets, computers, or online life storybooks. VR interventions (3 studies) [11, 18, 23] used head-mounted displays (HMD) to provide immersive reminiscence experiences, such as 360° videos of meaningful locations or interactive historical scenes.

Intervention intensity varied across studies. Program duration ranged from 2 to 24 weeks, with most interventions lasting from 5 to 9 weeks. Session frequency ranged from 0.5 to 2 sessions per week, most commonly twice weekly, and session duration ranged 10 to 90 min, with the majority of studies reporting sessions lasting from 30 to 60 min.

Among the RCTs, two studies [11, 21] used traditional RT as the comparison group, while five studies [16, 20, 24, 25, 28] used usual care as the control condition.

Change-from-baseline data were unavailable for most included randomized controlled trials. Baseline characteristics between intervention and control groups were generally comparable across all outcome domains, with no meaningful differences considered likely to influence the results; therefore, post-intervention values were used in the primary analyses. Baseline means and standard deviations are summarized in Additional File 1.

### Risk of Bias

Table 2A and 3B summarize the methodological quality of the included randomized and non-randomized studies, respectively.

Among the RCTs assessed using RoB 2 tool (Table 2A), five studies [11, 16, 20, 21, 24] were rated “some concerns” overall. These ratings were primarily driven by Domain 2 (deviations from intended interventions), reflecting the lack of participant and personnel blinding, which is inherently challenging in behavioral interventions and could have influenced participant engagement or facilitator behavior. Two studies [25, 28] were rated as “high risk” due to multiple issues, including absence of allocation concealment, lack of blinding, and the same researcher delivering the intervention and assessing outcomes. These issues increased the risk of selection, performance, and detection bias across multiple RoB domains.

For non-randomized studies, risk of bias was assessed using the MINORS tool (Table 3B). Most studies [17–19, 22, 23, 27] scored 11–12 out of a maximum of 16 points, indicating moderate methodological quality. Common limitations included the absence of sample size calculations and unblinded outcome assessment. Additional concerns included unclear prospective data collection and participant inclusion, limiting the overall validity. Only one study [26] was rated as low risk of bias (score = 15), reflecting more complete reporting and stronger methodological rigor across MINORS domains.

**Table 2:** Risk of Bias of RCTs using the RoB 2 tool

Study	D1	D2	D3	D4	D5	Overall
Perez et al. (2021) [16]	Low	Some concerns	Low	Low	Some concerns	Some concerns
Zhao and Zhang (2018) [28]	Some concerns	Some concerns	Low	Some concerns	Low	High
Manav et al. (2019) [25]	Some concerns	Some concerns	Low	Some concerns	Low	High
Yu et al. (2019) [24]	Low	Some concerns	Low	Low	Low	Some concerns
Moon and Park (2020) [21]	Low	Some concerns	Some concerns	Low	Low	Some concerns
Elfrink et al. (2021) [20]	Some concerns	Some concerns	Low	Low	Low	Some concerns
Tominari et al. (2021) [11]	Low	Some concerns	Low	Some concerns	Low	Some concerns

D1 = Bias arising from the randomization process, D2 = Bias due to deviations from intended interventions, D3 = Bias due to missing outcome data; D4 = Bias in measurement of the outcome; D5 = Bias in selection of the reported result

**Table 3:** Risk of Bias of non-randomized studies using MINORS tool

Study	(1)	(2)	(3)	(4)	(5)	(6)	(7)	(8)	Total score
Park and Moon (2022) [17]	2	1	2	2	1	2	2	0	12
Abdalrahim et al. (2022) [19]	2	1	2	2	1	2	1	0	11
Francis et al. (2020) [22]	2	1	2	2	1	2	1	0	11
Coelho et al. (2020) [23]	2	1	2	2	1	2	1	0	11
Astell et al. (2018) [26]	2	2	2	2	2	2	1	2	15
Subramaniam and Woods (2016) [27]	2	1	2	2	1	2	2	0	12
Huang and Yang (2022) [18]	2	1	2	2	1	2	1	0	11

MINORS assesses 8 domains, each scored 0 (not reported), 1 (inadequate), or 2 (adequate), maximum 16 points. Domains: (1): Clearly stated aim, (2) Inclusion of consecutive patients, (3) Prospective data collection, (4) Appropriate endpoints, (5) Unbiased endpoint assessment, (6) Adequate follow-up period, (7) Loss to follow-up < 5%, (8) Prospective sample size calculation

### Effects on cognition performance

#### RCTs assessing global cognition (MMSE)

**Fig. 2:**
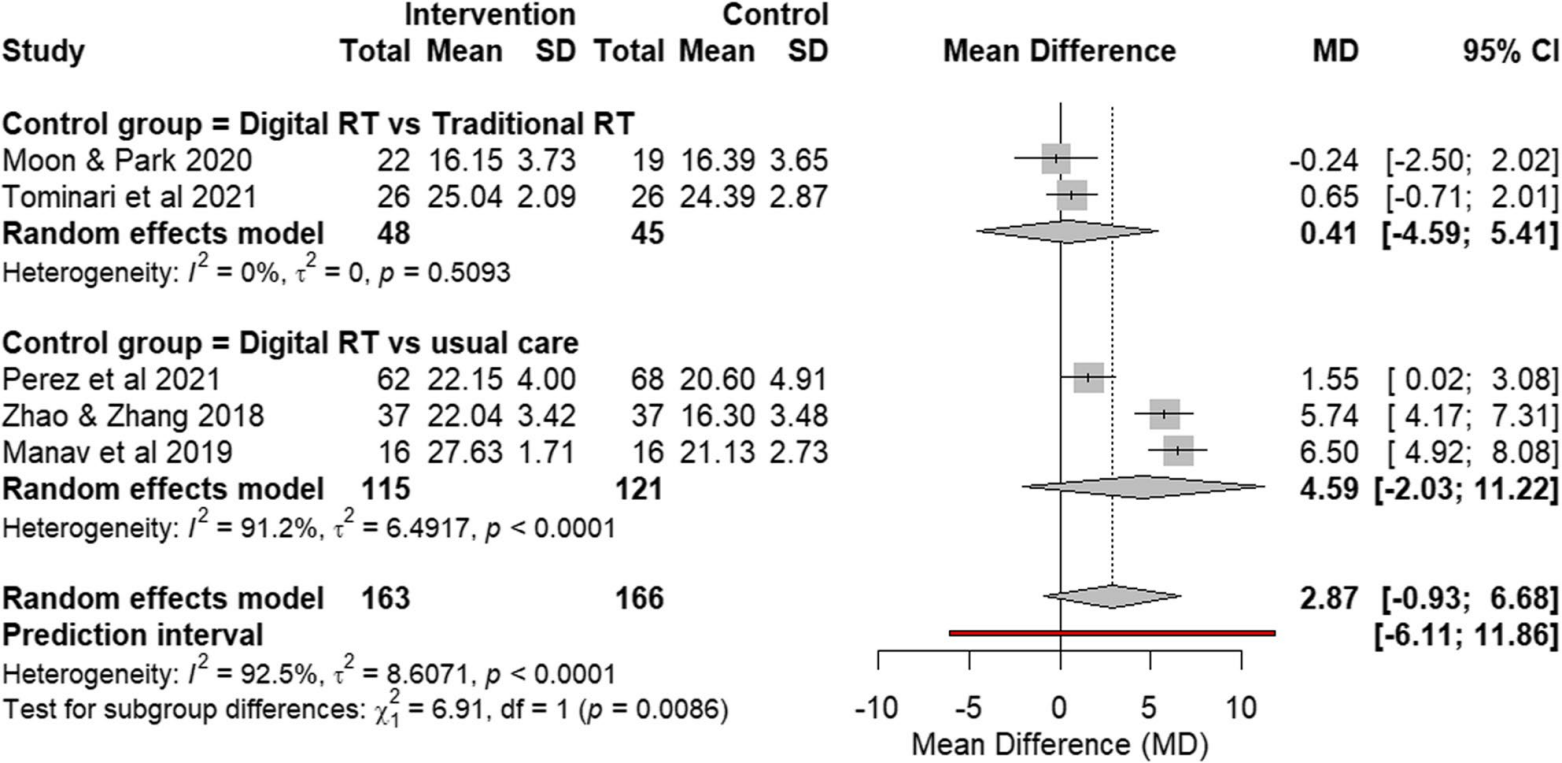
Forest plot of global cognition outcome (MMSE scores) from RCTs. Effect size: MD with 95% CI

Five RCTs [11, 16, 21, 25, 28] that assessed global cognition using MMSE were pooled. Post-intervention values were analyzed across all studies, with higher MMSE scores indicating better cognitive performance (Fig. 2).

##### Digital RT vs traditional RT

The subgroup analysis of two RCTs [11, 21] that compared digital RT with traditional RT as an active control found no significant difference between the two interventions. This indicates that digital RT was not superior to established, non-digital RT therapy for improving global cognition.

##### Digital RT vs usual care

Three RCTs [16, 25, 28] compared digital RT to a usual care control group. Although individual studies showed a tendency toward improvement, the findings within this subgroup did not demonstrate a significant overall effect, with the analysis being marked by substantial statistical heterogeneity (I² = 91.2%).

### Overall pooled effect and sensitivity analysis

The overall pooled effect across all five RCTs suggested no significant difference in global cognition. The clinical interpretability of this pooled result was notably limited by the high statistical heterogeneity. Consistent with this variability, the 95% prediction interval was wide, indicating that the effect of digital reminiscence therapy on cognition may vary substantially across different settings.

**Fig. 3:**
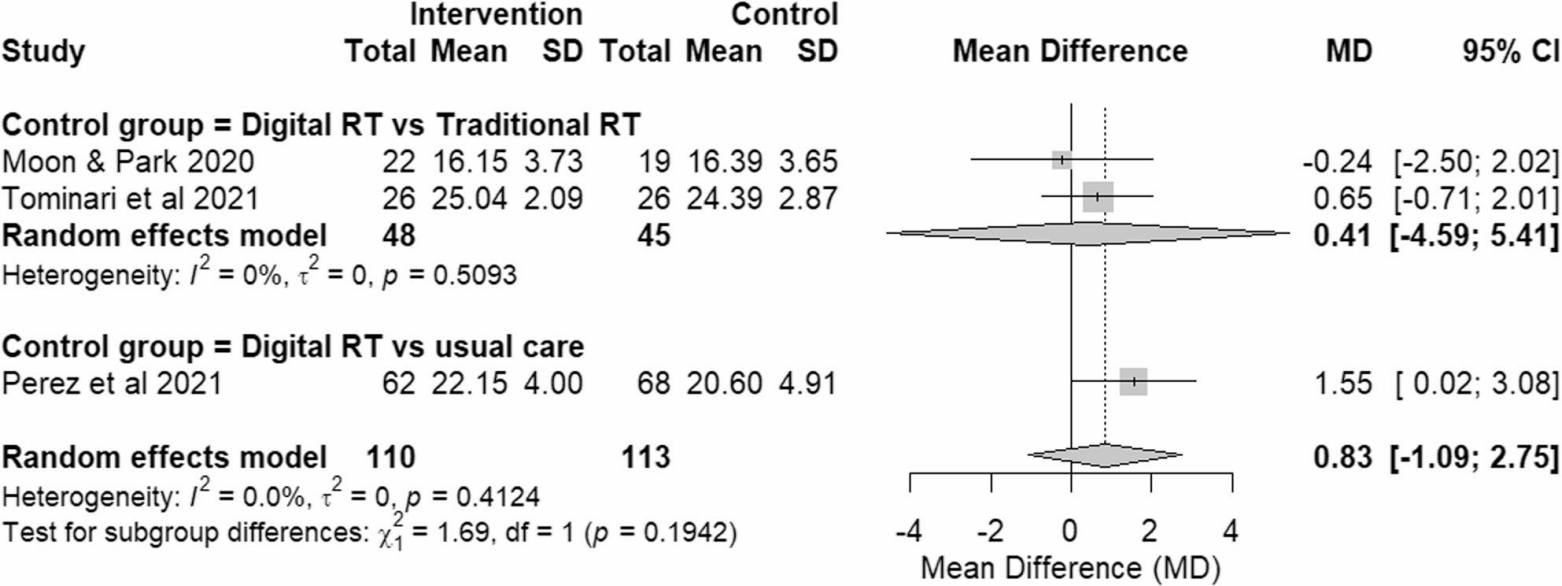
Sensitivity analysis of cognition outcomes from RCTs, excluding the high-risk of bias studies. Effect size: MD with 95% CI

A sensitivity analysis excluding the studies with high risk of bias [25, 28] showed low heterogeneity (I² = 0%), confirming that the variation in results was largely attributable to methodological limitations in those specific trials. However, the effect in low-risk of bias studies remained non-significant (Fig. 3).

Leave-one-out influence analyses further showed that omission of any single study did not materially alter the pooled effect estimate or the overall conclusion.

Subgroup analysis

**Fig. 4:**
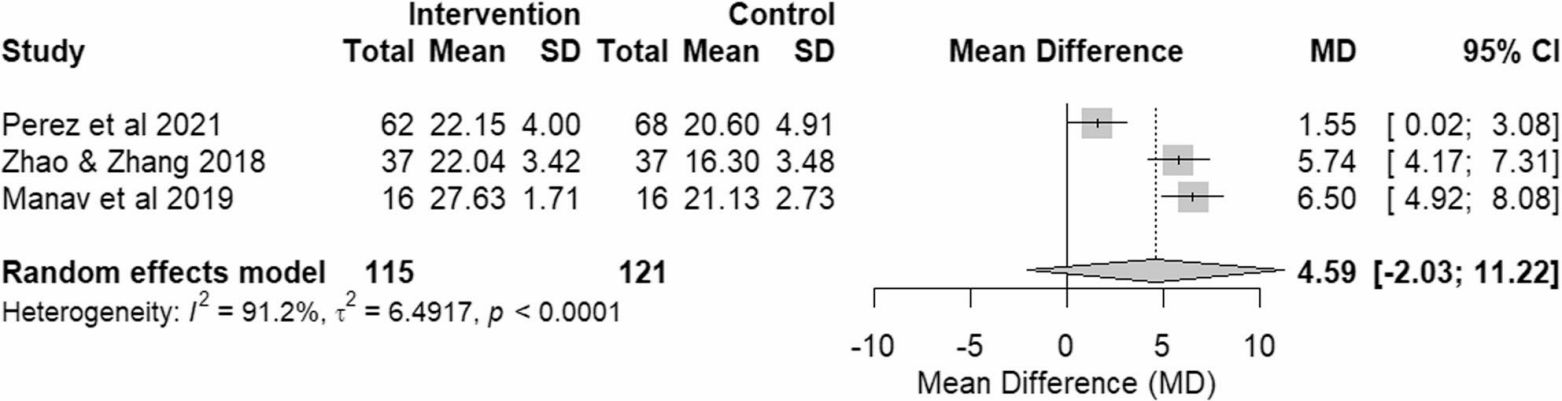
Forest plot of MMSE scores from RCTs with subgroup analysis of digital multimedia interventions. Effect size: MD with 95% CI

A pre-specified subgroup analysis was also conducted to explore the effect of different digital intervention types. The analysis of the three RCTs that utilized digital multimedia interventions showed no significant effect on global cognition (Fig. 4), while the other two digital formats (VR and interactive 2D platforms) could not be analyzed due to an insufficient number of studies.

Additional exploratory stratified analyses were performed to examine potential effect modification by personalization level and social engagement level. Neither analysis revealed statistically significant differences between subgroups, and confidence intervals were wide due to the limited number of studies per category (Additional File 2 and Additional File 3). Notably, the trial of Manav et al. [25] with a high level of social engagement, delivered in a group format, showed a numerically larger effect estimate compared with studies involving moderate social engagement through interaction with caregivers, volunteers, or facilitators. Similarly, the study by Zhao and Zhang [28], which employed a high level of intervention personalization, showed a larger effect estimate relative to studies using less personalized content. However, this observation was based on a single study and should be interpreted cautiously.

### RCTs assessing memory function

**Fig. 5:**
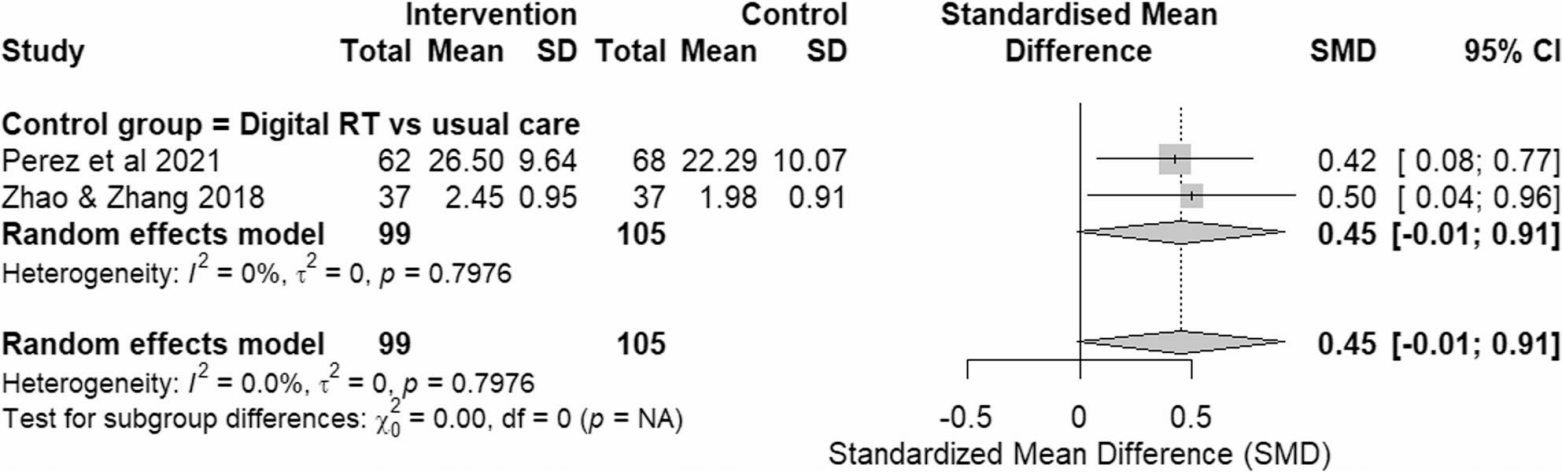
Forest plot of memory function outcomes from RCTs. Effect size: SMD with 95% CI

Two RCTs [16, 28] additionally evaluated memory function outcomes, with Perez et al. [16] employing MAT and Zhao and Zhang [28] using the WHO-UCLA AVLT; in both instruments, higher scores indicate better memory performance. Both studies used usual care as the control group. For the WHO-UCLA AVLT, the delayed recall subscale was selected for analysis, as it most directly reflects episodic memory function. Meta-analysis using SMD indicated no significant overall effect of digital RT on memory (Fig. 5).

### One-arm pre-post studies assessing cognition performance

Four one-arm pre-post studies [18, 19, 26, 27] evaluated cognitive outcomes, yielding mixed results. Subramaniam et al. [27] used a digital life story book (movie format with music, narration and video) and reported improved personal semantic memory on the Autobiographical Memory Interview-Extended, Personal Semantic Schedule (AMI-E PSS; +8.92) but a slight decline in episodic memory on the Autobiographical Memory Interview-Extended, Autobiographical Incidents Schedule (AMI-E AIS; -1.5). Huang et al. [18] applied immersive VR via HMD with interactive historic scenes, finding no significant change on MMSE (-0.45) or Cognitive Abilities Screening Instrument (CASI; -1.01). In contrast, Abdalrahim et al. [19] delivered 10 sessions on digital RT using a touch-screen app with photos, videos and audio, and observed significant gains (SLUMS; +3.7, p < .001) after 5 weeks. Astell et al. [26] employed the CIRCA program (Computer Interactive Reminiscence and Conversation Aid) and reported improvements on the Addenbrooke’s Cognitive Examination-III (ACE-III; +2.24, p = .007) with further gains at 3 months (+3.62, p = .01).

### Effects on mood domain

#### RCTs assessing mood domain

**Fig. 6:**
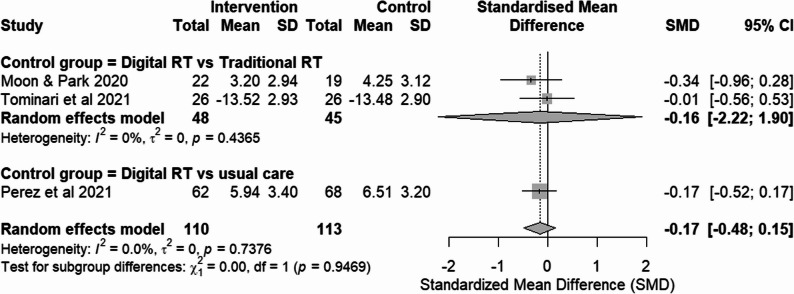
Forest plot of mood outcomes from RCTs

Three RCTs [11, 16, 21] evaluated depressive symptoms using different scales: the Cornell Scale for Depression in Dementia (CSDD), the Revised Philadelphia Geriatric Center (PGC) Morale Scale and the Geriatric Depression Scale 15 (GDS-15). For the CSDD and GDS-15, higher scores indicate greater depressive symptom severity, meanwhile, for PGC Morale Scale, higher scores indicate better mood. To ensure consistent interpretation, scores from the PGC Morale Scale used in Tominari et al. [11] were directionally adjusted prior to pooling. Post-intervention values were analyzed for all studies. Effect sizes were calculated as SMD (Fig. 6).

##### Digital RT vs traditional RT

Subgroup analysis of 2 studies [11, 21] showed no significant effect with low heterogeneity. This suggests that digital RT was not superior to traditional RT in improving depressive symptoms among participants with dementia.

##### Digital RT vs usual care

Subgroup analysis of studies using usual care as the comparator was not performed due to the availability of only one study [16]. In this study, the result was not significant, although it showed a trend towards improvement in depressive symptoms.

### Overall pooled effect

The pooled SMD of all RCTs assessing mood domain showed no significant effect, with low heterogeneity (I² = 0%, p = .74).

Leave-one-out influence analyses indicated that exclusion of any single study did not materially change the pooled estimate or the overall conclusion.

### Subgroup analysis

Subgroup analyses for the mood domain were limited by the small number of studies. Stratification by digital format was not feasible because each included RCT employed a different intervention format. Similarly, analyses by social engagement level were not conducted, as all mood interventions involved facilitated delivery with comparable levels of human support.

Exploratory stratified analysis by personalization level did not reveal statistically significant differences between subgroups (Additional File 4). However, the study by Moon and Park [21], which incorporated a high degree of autobiographical personalization based on structured interviews with family members, demonstrated the largest numerical improvement in mood outcomes, whereas the other two studies employed generic reminiscence content relevant to participants’ historical or cultural backgrounds. This pattern was driven by a single study and should therefore be interpreted cautiously.

### Other RCTs results not pooled in meta-analysis

In addition, Tominari et al. [11], who compared digital RT with traditional RT, also measured mood with the Multidimensional Observation Scale for Elderly Subjects (MOSES) depression subscale, reporting MD = 0.50 (p = .20). However, this outcome was not included in the meta-analysis, as the MOSES scale is less specific for depressive symptoms than the PGC.

Study of Yu et al. [24] evaluated mood using the Alzheimer’s Disease and Related Dementias Mood Scale (AMS), but the results were not pooled due to lack of reported data to calculate SMD. The study reported no significant group differences between digital RT and usual care at 12 weeks, although modest improvements were noted in “contented” and “spirited” mood within the individual intervention arm.

### One-arm pre-post studies assessing mood domain

Three one-arm pre-post studies found reductions in depressive symptoms. Abdalrahim et al. [19] found significant decreases in both depression (-3.7, SD = 2.6) and anxiety (-4.4, SD = 2.9) in the Arabic version Hospital Anxiety and Depression Scale (HADS) (both *p* < .001). Subramaniam et al. [27] observed a 1.84-point reduction on GDS-12R. while Huang et al. [18] reported a MD of -3.00 on the Center for Epidemiologic Studies Depression Scale (CES-D).

### Effects on BPSD domain

#### RCTs assessing BPSD domain

**Fig. 7:**
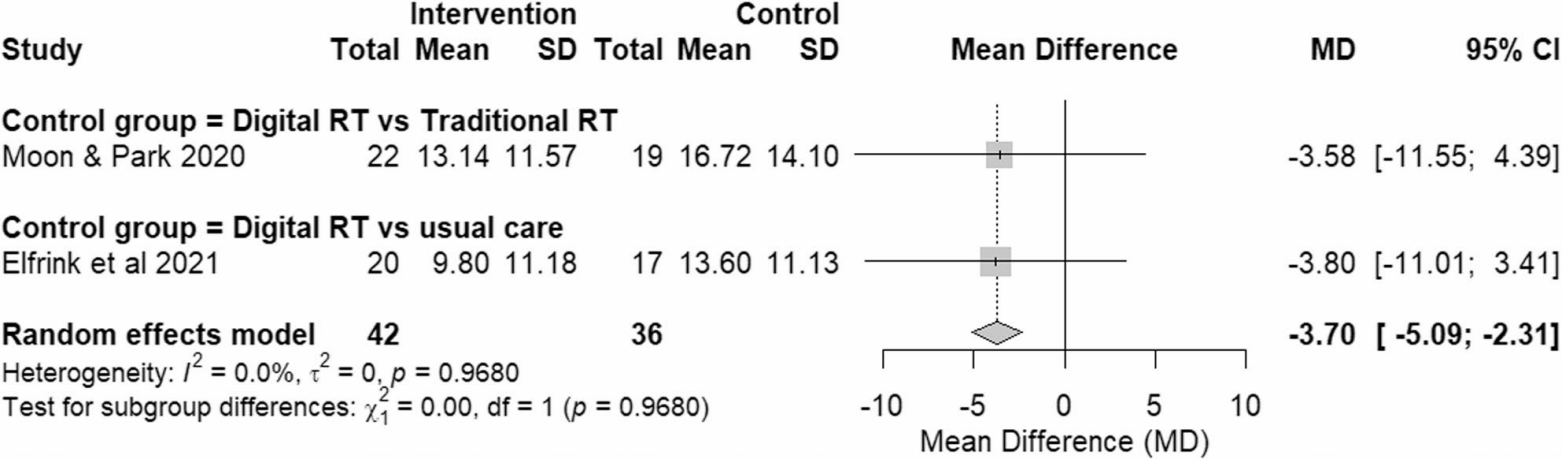
Forest plot of BPSD domain (NPI scores) from RCTs. Effect size: MD with 95% CI

Two RCTs [20, 21] evaluated BPSD using NPI, with higher scores indicating more severe symptoms, and were pooled using MD. Post-intervention values were analyzed for all studies (Fig. 7). For the study by Elfrink et al. [20], the SD was calculated from the reported standard error and sample size to enable inclusion in the meta-analysis.

Subgroup analysis based on comparator types was not statistically feasible due to the availability of only one study in each subgroup. Individually, both studies showed a tendency towards reducing the NPI score, indicating fewer behavioral and psychological symptoms, but neither achieved statistically significant results.

### Overall pooled effect

When pooled together, digital RT demonstrated a significant overall reduction in symptoms (MD = -3.70, 95% CI from -5.09 to -2.31), with low evidence of heterogeneity (I² = 0%, p = .97). However, this statistically significant pooled effect is based on only two RCTs using a single instrument (NPI), and other RCTs assessing BPSD with different measures did not demonstrate significant benefits.

### Subgroup analysis

Subgroup analyses for the BPSD domain were not feasible due to the limited number of studies, with only one study available in each subgroup category. Both included trials employed interactive 2D platform-based interventions, precluding comparison by digital format. With respect to delivery characteristics, the study by Moon and Park [21] was characterized by a moderate level of social engagement, whereas Elfrink et al. [20] involved a high level of social engagement. Both studies incorporated a high degree of content personalization.

### Other RCTs results not pooled in meta-analysis

Two other RCTs [11, 24] were not pooled into the meta-analysis due to incompatible outcome measures. Specifically, Yu et al. [24] used NPI Questionaire (NPI-Q) and the hostile mood subscale of the AMS, finding no group differences at 6 or 12 weeks between digital RT and usual care. Tominari et al. [11] assessed irritability using the MOSES irritability subscale, also reporting no significant effect (MD = 0.42, 95% CI -0.20 to 1.05, *p* = .18) between digital RT and traditional RT.

### One-arm pre-post studies assessing BPSD domain

Two one-arm pre-post studies [22, 23] assessed BPSD using NPI. Francis et al. [22] delivered a 30-minute personalized biographical film and reported a significant reduction in BPSD symptoms with a mean decrease of 14.71 (*p* = .042). In contrast, Coelho et al. [23] used personalized 360° videos of meaningful locations via VR headsets and observed no significant change in NPI scores following the intervention.

### Effects on communication and engagement

#### RCTs assessing Communication and Engagement

Four RCTs [11, 20, 21, 25] assessed the communication and engagement domain using instruments that capture related constructs of apathy, social participation, and interpersonal involvement. These included the MOSES Withdrawal subscale, which reflects social withdrawal and reduced interaction; the NPI Apathy subscale, which measures motivational and engagement-related symptoms; the Engagement of a Person with Dementia Scale (EPWDS), which assesses observable participation and involvement in activities; and the Apathy Rating Scale (ARS) - Self-assessment version, which evaluates self-perceived motivation and interest.

**Fig. 8:**
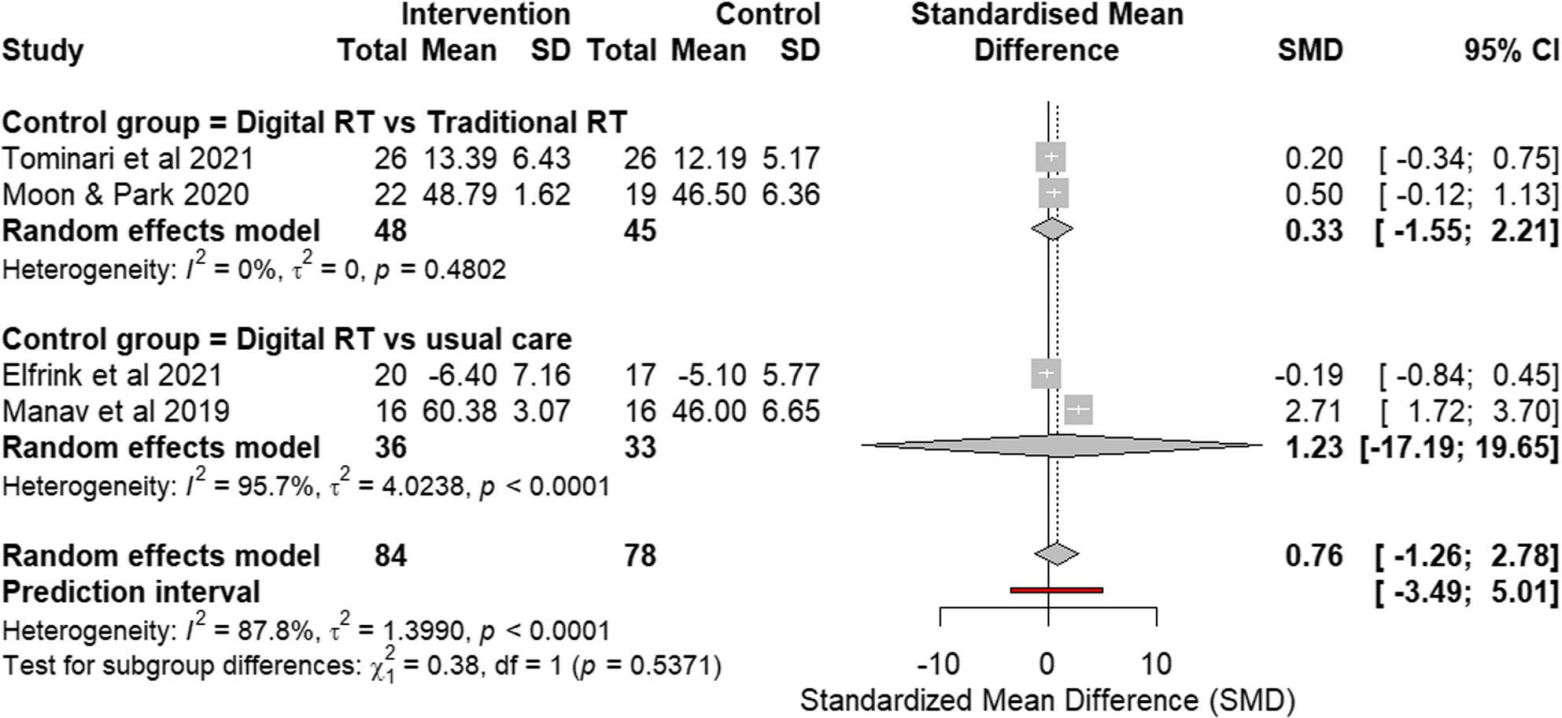
Forest plot of communication and engagement outcomes from RCTs. Effect size: SMD with 95% CI

Higher scores in MOSES Withdrawal subscale, EPWDS and ARS indicate improvement in symptoms. Higher score in NPI Apathy subscale indicates more apathy symptoms and were therefore directionally reversed in this analysis to ensure consistent interpretation across measures. Post-intervention values were analyzed for all studies (Fig. 8). For the study by Elfrink et al. [20], SD were calculated from the reported standard errors and sample sizes to allow inclusion in the pooled analysis.

#### Digital RT vs. traditional RT

Subgroup analysis of studies comparing digital RT with traditional RT showed no significant effects and was characterized by high heterogeneity. Although one study [21] showed significant effect on EPWDS, the analysis suggests digital RT may not be superior to traditional RT in terms of reducing apathy and encouraging communication and engagement in dementia patients.

#### Digital RT vs. usual care

Subgroup analysis of studies comparing digital RT with usual care similarly showed no significant effect, suffering from substantial statistical heterogeneity. One study [20] reported worsening apathy using NPI Apathy subscale, while the other study [25] showed reduction in apathy symptom using ARS.

### Overall pooled effect and sensitivity analysis

The pooled SMD across all four RCTs indicated no significant overall effect. The analysis was marked by substantial heterogeneity (Fig. 8). Consistent with this variability, the 95% prediction interval was wide, indicating that the effect of digital reminiscence therapy may vary considerably across different future settings.

**Fig. 9:**
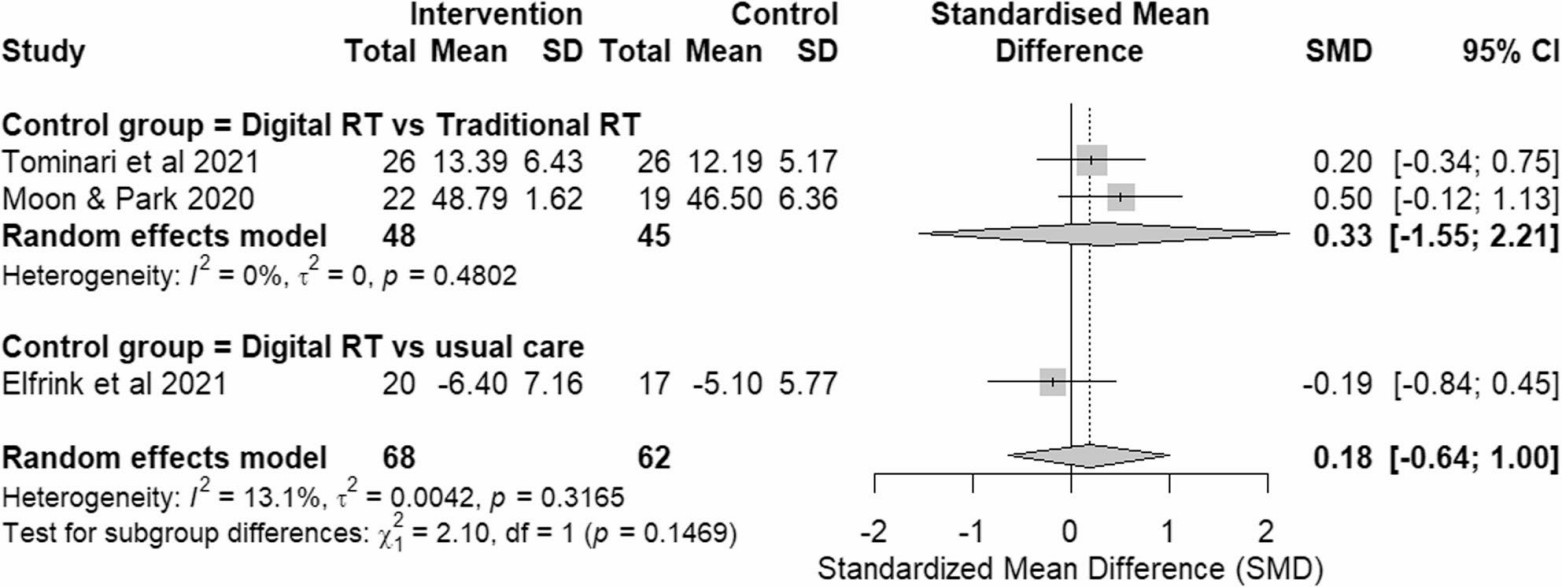
Sensitivity analysis of communication and engagement outcomes from RCTs, excluding the high-risk of bias study. Effect size: SMD with 95% CI

A sensitivity analysis excluding the study with high risk of bias [25] substantially reduced heterogeneity (I² = 13.1%), indicating that between-study variability was largely driven by methodological limitations in this trial. Despite this reduction in heterogeneity, the pooled effect estimate among studies at low risk of bias remained non-significant (Fig. 9).

Leave-one-out influence analyses indicated that exclusion of any single study did not materially change the pooled estimate or the overall conclusion.

### Subgroup analysis

**Fig. 10:**
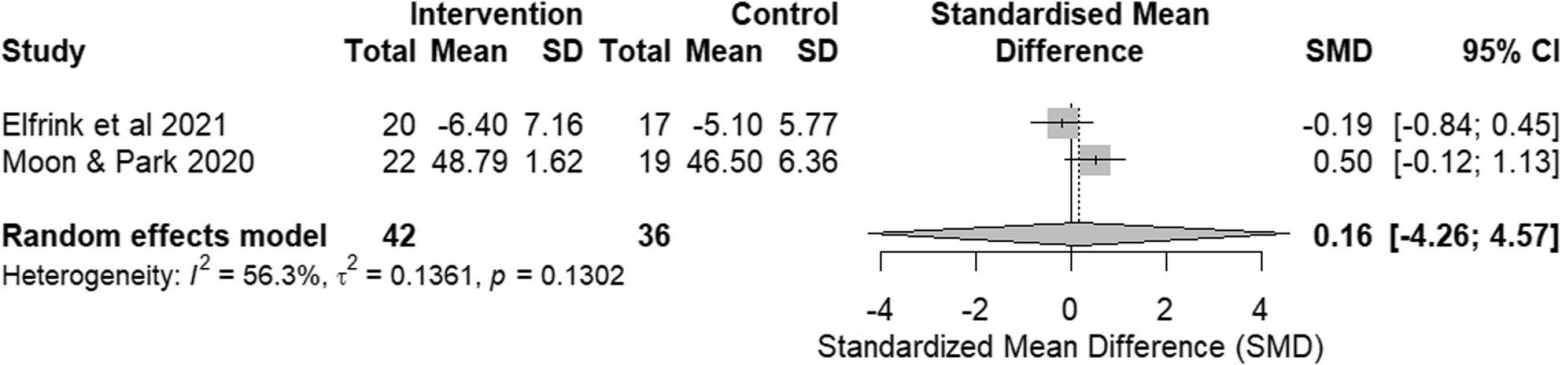
Forest plot of communication and engagement outcomes with subgroup analysis of interactive 2D platforms. Effect size: SMD with 95% CI

Subgroup analysis of interactive 2D platforms for the communication and engagement domain showed no significant effect (Fig. 10), and this analysis was also characterized by high heterogeneity. The other two formats (VR and digital multimedia) could not be analyzed due to insufficient studies.

Additional exploratory stratified analyses based on personalization level and social engagement level likewise did not demonstrate statistically significant subgroup differences (Additional File 5 and Additional File 6). However, the study by Manav et al. [25], which employed a group-based format corresponding to a high level of social engagement, showed the largest numerical improvement in apathy outcomes compared with studies delivered in individual-based formats. This observation was driven by a single study and should therefore be interpreted cautiously.

#### Other RCTs results not pooled in meta-analysis

Additionally, another RCT was not pooled into the meta-analysis due to lack of data for SMD calculation. Yu et al. [24] evaluated engagement using the Pleasant Events Schedule - Alzheimer’s Disease (PES-AD), where the digital RT arm outperformed both group RT (*p* = .017) and usual care (*p* = .005) at 6 weeks, though gains were not sustained at 12 weeks. AMS results showed a trend toward reduced apathy at 12 weeks in the group RT arm (*p* = .051), suggesting possible long-term motivational benefits.

### One-arm pre-post studies assessing communication and engagement

Two one-arm pre-post studies [17, 19] reported significant improvements. Abdalrahim et al. [19] and Park and Moon [17] (a secondary analysis of Moon and Park [21], using an Android tablet app with personalized materials based on family input) both used Holden Communication Scale (HCS), finding reduced scores (indicating better communication), with Abdalrahim et al. [19] reporting mean = 6.2 (SD = 4.2, *p* < .001) and Park and Moon [17] F = 3.56 (*p* = .033).

### Quality of life domain

#### RCTs assessing quality of life domain

Two RCTs [16, 24] reported QOL outcomes but were not pooled for meta-analysis due to use of different tools and insufficient data for SMD calculation. Yu et al. [24] used the Cantril QOL Ladder, found a non-significant improvement. In contrast, Perez et al. [16], using the Quality of Life in Alzheimer’s Disease (QOL-AD) scale, reported significant gains in the intervention group (*p* = .009), whereas scores in the control group declined (*p* = .044).

### One-arm pre-post studies assessing quality of life domain

Four one-arm pre-post studies [19, 22, 23, 27] evaluated QOL, with mixed results. Francis et al. [22] and Subramaniam et al. [27] both using the QOL-AD, reported non-significant improvements. Coelho et al. [23] used European Health Interview Survey - Quality of Life 8-item index (EUROHIS-QOL-8) and similarly found no significant improvement. In contrast, Abdalrahim et al. [19] reported a significant increase in Older People’s Quality of Life questionnaire, Brief 13-item version (OPQOL-Brief-13) (+ 6.2, SD 4.8, *p* < .001) after five weeks.

### Intervention dose characteristics

Intervention dose characteristics are summarized descriptively in Additional File 7; reporting of intervention intensity varied substantially across studies, with some trials specifying fixed session numbers and durations and others reporting ranges or estimated exposure, precluding formal dose-response analysis, and therefore the table should be interpreted as a descriptive summary rather than a dose-response model.

### Summary of pooled effects of RCTs across all domains

Using the GRADE approach, certainty of evidence was assessed for each outcome domain and explicitly aligned with the analytic findings. Cognitive, mood, BPSD, and communication/engagement outcomes from RCTs were rated as having moderate certainty. In these domains, no serious risk of bias or inconsistency was identified, as risk-of-bias-restricted sensitivity analyses did not materially change pooled estimates and, where applicable, reduced heterogeneity. However, certainty was downgraded by one level for imprecision due to wide confidence intervals and modest total sample sizes. Specifically, risk of bias was not judged to be serious because exclusion of studies at high risk of bias did not meaningfully alter the magnitude or direction of effects, whereas imprecision remained a concern due to limited sample sizes and confidence intervals spanning clinically relevant ranges.

The memory domain was rated as having low certainty, reflecting the inclusion of only two trials, one of which was at high risk of bias, and the inability to conduct risk-of-bias-restricted analyses, together with substantial imprecision. No downgrading for indirectness was applied across outcomes, as all included studies evaluated populations, interventions, and outcomes directly relevant to digital reminiscence therapy using validated assessment tools (Table 4).

**Table 4:** Summary of meta-analysis results and GRADE assessment

Study design/ Analysis	No. of studies	No. of participants	Pooled effect (95% CI)	I² (%)	Interpretation	GRADE Assessment
RCTs for Global cognition (MMSE)	5	329	MD = 2.87 [-0.93; 6.68]	92.5%	Not significant, high heterogeneity	Moderate
RCTs for Memory subdomain	2	204	SMD = 0.45 [-0.01; 0.91]	0%	Not significant	Low
RCTs for Mood domain	3	223	SMD = -0.17 [-0.48; 0.15]	0%	Not significant	Moderate
RCTs for BPSD domain (NPI)	2	78	MD = -3.70 [-5.09; -2.31]	0%	Significant improvement	Moderate
RCTs for communication and engagement	4	162	SMD = 0.76 [-1.26; 2.78]	87.8%	Not significant, high heterogeneity	Moderate

## Discussion

This systematic review and meta-analysis evaluated the efficacy of digital RT for people with cognitive impairment across multiple outcome domains. Among RCTs, significant improvement was observed only in the pooled result for BPSD (MD = -3.70, NPI), while effects on cognition, mood, and QOL were non-significant. Evidence from one-arm pre-post studies suggested possible benefits across several domains, but methodological limitations, particularly small sample sizes and lack of control groups, limited these findings to narrative synthesis.

According to the GRADE assessments, the certainty of evidence ranged from moderate (for global cognition, mood, BPSD, communication and engagement) to low (memory), indicating cautious interpretation. Heterogeneity likely arose from differences in intervention design, comparators (traditional RT or usual care), and outcome measures. Digital RT was delivered via VR, mobile/tablet applications, or digital multimedia, each providing varying stimulation and engagement. In addition, interventions differed in the degree of autobiographical personalization and social engagement, which may influence emotional and cognitive responses and contribute to variability in effects. Comparisons included usual care or traditional RT, with the latter potentially attenuating effect sizes given its established benefits. Despite using SMDs, outcome variability across scales contributed to residual heterogeneity. These factors highlight the need for adequately powered, multicenter RCTs with standardized protocols and outcome measures.

The pooled reduction in BPSD symptoms, derived from two studies with different comparator groups, suggests that digital RT may modestly reduce behavioral symptoms in dementia. Low statistical heterogeneity indicated consistency across studies, and the significant pooled result may reflect increased statistical power from combining trials with a similar trend. However, this finding was based on only two trials with small sample sizes and different types of comparators, therefore, must be interpreted with considerable caution and restraint.

The observed benefits of digital RT on BPSD may have stemmed from shared features across studies, particularly high levels of content personalization and social engagement as defined in our taxonomy. Most interventions contributing to favorable outcomes were classified as high-personalization, incorporating individualized materials, such as digital life story books [20], biographical films [22] or media from family interviews [21]. Developed with caregivers or volunteers, these materials reflected each participant’s personal history, making them emotionally salient and meaningful. By stimulating autobiographical memory, personalization reinforced self-continuity and evoked positive emotions. In addition, several effective interventions were categorized as moderate- to high-engagement formats, being delivered in one-on-one [21] or in small groups [20, 22], fostering meaningful social interaction and engagement. Consistent with this, subgroup and stratified analyses presented in the supplementary forest plots suggested more favorable effects in interventions characterized by higher engagement and personalization, although the limited number of studies precludes firm conclusions. Social contact can reduce loneliness and isolation, key drivers of behavioral disturbances [29, 30]. A supportive relational environment may thus amplify the effects of personalized content and improve BPSD.

Similar mechanisms have been reported in non-dementia populations, where personalized, emotionally meaningful interventions promote psychological well-being [31–33]. This suggests that digital RT leverages general psychological processes - emotional salience, identity reinforcement, and social connection - that underpin behavioral benefits across populations.

Results from global cognition, memory, mood, communication and engagement were all statistically non-significant, with substantial heterogeneity and moderate to low certainty. The high inconsistency across trials, even in pre-specified subgroups comparing different comparators, may imply that the heterogeneity was primarily driven by clinical diversity, such as duration or frequency of the sessions, the delivery methods, or outcome scales, rather than merely the difference between comparators. Consequently, no firm conclusions can be drawn for these domains.

A key strength of this review is its exclusive focus on digital RT, providing a timely synthesis of this emerging non-pharmacological approach. Unlike previous reviews [8, 12], limited to traditional RT or certain domains of digital RT, this study evaluated digital modalities across cognition, mood, BPSD, QOL, communication and engagement, while meticulously analyzing the underlying methodological factors, such as heterogeneity in intervention characteristics and control group composition, that limited the certainty of the overall evidence. Furthermore, our study provided quantitative evidence of possible underlying mechanisms of effect on BPSD domain. By analyzing possible underlying mechanisms, such as personalization and social interaction, this review provides insight into why digital RT may be effective, supporting clinical hypothesis generation. Use of the Revised Cochrane Risk of Bias Tool (RoB 2), the Methodological Index for Non-Randomized Studies (MINORS), and RCT-based meta-analyses ensured methodological rigor, while narrative synthesis of one-arm studies added context by capturing feasibility and emerging evidence. This level of focused detail is essential because it provides an in-depth review of digital RT.

Several limitations should be acknowledged. Only seven RCTs were included, most with small sample sizes and variable outcome measures, contributing to heterogeneity and downgrades in GRADE certainty. Although memory within cognition domain was expected to be directly affected, only two RCTs assessed this domain, limiting broader conclusions. Subgroup analyses by intervention format, personalization level, and social engagement level were feasible for some outcome domains but not others; however, the small number of studies within most subgroups precluded reliable interpretation or robust comparisons across categories. Furthermore, subgroup analyses by dementia severity or subtype were not feasible, as the included studies did not explicitly stratify or report outcomes by these categories. Assessment of intervention intensity was limited by variable and incomplete reporting of dose, which hindered formal dose-response analysis. Finally, the limited number of included trials in several domains precluded formal assessment of publication bias and raises the possibility of small-study effects, which may influence the magnitude of observed effects.

Digital RT may offer small to moderate benefits in reducing BPSD compared with mixed control groups. While the pooled result reached statistical significance and moderate certainty, the evidence base remains limited and heterogeneous. Mechanisms such as personalization and social interaction likely contribute to these benefits by addressing emotional and social needs in dementia care. However, evidence for cognition, mood, and other domains remains inconclusive. Future research should prioritize large, multicenter randomized controlled trials using standardized digital reminiscence therapy protocols with clearly defined and consistently reported dosage parameters. Interventions should be systematically characterized by delivery format, level of autobiographical personalization, and degree of social engagement to enable meaningful comparisons across studies. Future trials should employ validated outcome measures and include direct comparisons with both traditional reminiscence therapy and usual care. Stratified analyses by dementia stage or subtype, as well as by key intervention features such as personalization and social facilitation, are needed to clarify which digital RT formats are most effective and for whom.

## Supplementary Information

Additional File 1: Baseline outcome scores by arm for meta-analyzed outcomes.

Additional File 2: Forest plot of MMSE scores from RCTs with subgroup analysis of personalization level.

Additional File 3: Forest plot of MMSE outcomes with subgroup analysis of social engagement level.

Additional File 4: Forest plot of mood domain with subgroup analysis of personalization level.

Additional File 5: Forest plot of communication and engagement outcomes with subgroup analysis of personalization level.

Additional File 6: Forest plot of communication and engagement outcomes with subgroup analysis of social engagement level.

Additional File 7: Descriptive summary of intervention intensity (dose).

## Data Availability

All data analyzed during this study are included in this article.

## Abbreviations

AD: Alzheimer’s disease
AMS: Alzheimer’s Disease and Related Dementias Mood Scale
AVLT: WHO–UCLA Auditory Verbal Learning Test
BPSD: Behavioral and psychological symptoms of dementia
CI: Confidence interval
GDS: Geriatric Depression Scale
GRADE: Grading of Recommendations Assessment, Development and Evaluation
HMD: Head–Mounted Displays
MAT: Memory Alteration Test
MCI: Mild cognitive impairment
MD: Mean difference
MINORS: Methodological Index for Non–Randomized Studies
MMSE: Mini–Mental State Examination
MOSES: Multidimensional Observation Scale for Elderly Subjects
NPI: Neuropsychiatric Inventory
PGC: Revised Philadelphia Geriatric Center
QOL: Quality of life
RCT: Randomized controlled trial
RoB 2: Revised Cochrane Risk of Bias Tool
RT: Reminiscence therapy
SD: Standard deviation
SMD: Standardized mean difference
VR: Virtual reality
