# Supplementary material for: Digital reminiscence therapy in dementia care: a systematic review and meta-analysis
Source: BMC Neurol. 2026 Mar 25;26:296. doi: 10.1186/s12883-026-04759-y (PMC13137691; doi:10.1186/s12883-026-04759-y)
Supplement: Supplementary file 1 — Additional File 1: Baseline outcome scores by arm for meta-analyzed outcomes. [file 12883_2026_4759_MOESM1_ESM.pdf]

Additional File 1: Baseline outcome scores by arm for meta-analyzed outcomes.

A. Cognition Performance

Global cognition

Study	Instrument	Control (mean ± SD)	Digital RT (mean ± SD)
Moon & Park 2020	MMSE	15.11 ± 3.62	14.73 ± 4.04
Manav et al 2019	MMSE	21.44 ± 1.09	21.69 ± 1.14
Tominari et al 2021	MMSE	23.69 ± 1.38	23.65 ± 1.57
Zhao & Zhang 2018	MMSE	14.98 ± 3.08	14.34 ± 3.26
Perez et al 2021	MMSE	21.15 ± 3.38	21.05 ± 3.82

Memory

Study	Instrument	Control (mean ± SD)	Digital RT (mean ± SD)
Zhao & Zhang 2018	WHO–UCLA AVLT	1.85 ± 1.13	1.64 ± 1.13
Perez et al 2021	MAT	22.57 ± 8.9	23.45 ± 10

B. Mood

Study	Instrument	Control (mean ± SD)	Digital RT (mean ± SD)
Moon & Park 2020	CSDD	4.42 ± 3.52	6.14 ± 4.78
Tominari et al 2021	Revised PGC morale scale score	14.2 ± 2.75	12.56 ± 3.9
Perez et al 2021	GDS-15	6.44 ± 3.56	6.05 ± 3.37

C. BPSD

Study	Instrument	Control (mean ± SD)	Digital RT (mean ± SD)
Moon & Park 2020	NPI	17.95 ± 14.76	16.24 ± 10.1
Elfrink et al 2021	NPI	9.8 (SE = 2.7)	8.7 (SE = 2.4)

D. Communication and Engagement

Study	Instrument	Control (mean ± SD)	Digital RT (mean ± SD)
Moon & Park 2020	EPWDS	47.67 ± 2.35	45.05 ± 3.59
Manav et al 2019	ARS	49.19 ± 4.39	51.75 ± 4.96
Tominari et al 2021	MOSES Withdrawal	13.12 ± 6.01	13.54 ± 6.08
Elfrink et al 2021	NPI Apathy	3.5 (SE = 1.4)	5.1 (SE = 1.5)
