# Supplementary material for: Digital reminiscence therapy in dementia care: a systematic review and meta-analysis
Source: BMC Neurol. 2026 Mar 25;26:296. doi: 10.1186/s12883-026-04759-y (PMC13137691; doi:10.1186/s12883-026-04759-y)
Supplement: Supplementary file 2 — Additional File 2: Forest plot of MMSE scores from RCTs with subgroup analysis of personalization level. [file 12883_2026_4759_MOESM2_ESM.pdf]

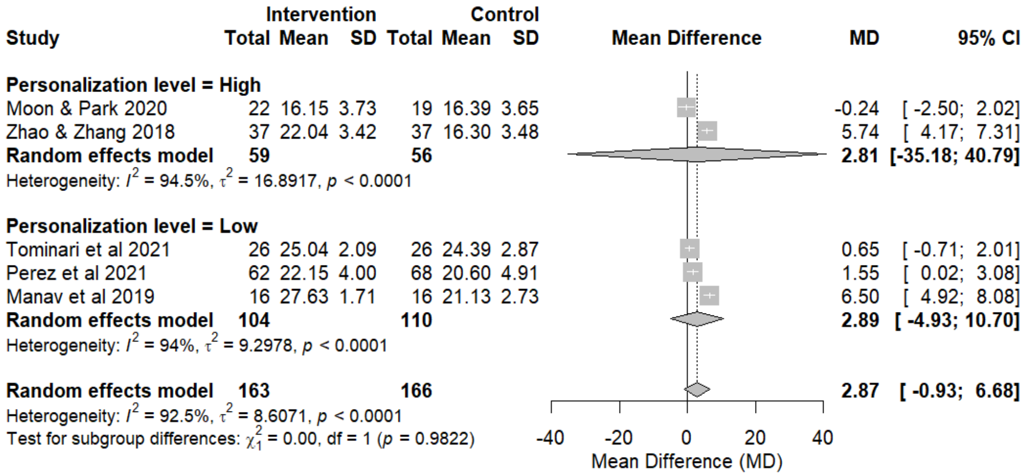

Additional File 2: Forest plot of MMSE scores from RCTs with subgroup analysis of personalization level.

Effect size: MD with 95% CI.
