# Supplementary material for: Digital reminiscence therapy in dementia care: a systematic review and meta-analysis
Source: BMC Neurol. 2026 Mar 25;26:296. doi: 10.1186/s12883-026-04759-y (PMC13137691; doi:10.1186/s12883-026-04759-y)
Supplement: Supplementary file 6 — Additional File 6: Forest plot of communication and engagement outcomes with subgroup analysis of social engagement level. [file 12883_2026_4759_MOESM6_ESM.pdf]

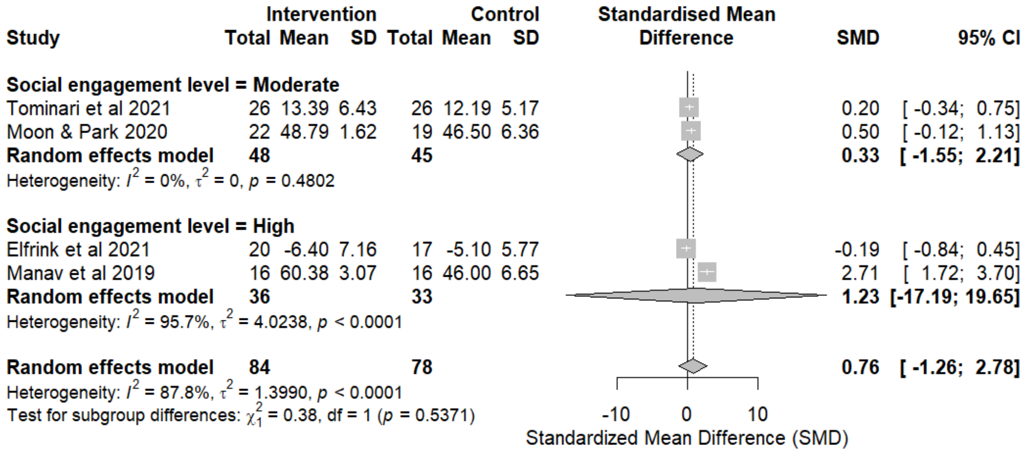

Additional File 6: Forest plot of communication and engagement outcomes with subgroup analysis of social engagement level

Effect size: SMD with 95% CI.
