# Supplementary material for: Digital reminiscence therapy in dementia care: a systematic review and meta-analysis
Source: BMC Neurol. 2026 Mar 25;26:296. doi: 10.1186/s12883-026-04759-y (PMC13137691; doi:10.1186/s12883-026-04759-y)
Supplement: Supplementary file 7 — Additional File 7: Descriptive summary of intervention intensity (dose). [file 12883_2026_4759_MOESM7_ESM.pdf]

Additional File 7: Descriptive summary of intervention intensity (dose)

Study	Number of Sessions	Duration per session	Total dose (minutes)	Notes on dose estimation
**Moon & Park, 2020**	2/week × 4 weeks (8 sessions)	30 min	240	
**Tominari et al., 2021**	1/week × 8 weeks (8 sessions)	30-45 min	300	Midpoint (37.5 min) used
**Elfrink et al., 2021**	5 sessions over 8–10 weeks	60-120 min	450	Midpoint (90 min) used
**Yu et al., 2019**	2/week × 6 weeks (guided) + encouraged self-play x 6 weeks	Not specified	Not estimable	Not estimable
**Manav et al., 2019**	1/week × 12 weeks (12 sessions)	60 min	720	
**Pérez-Sáez et al., 2021**	2/week × 13 weeks (26 sessions)	50 min	1300	
**Zhao & Zhang, 2018**	2/week × 4 weeks (8 sessions)	40–60 min	400	Midpoint (50 min) used
